# Epigenetic changes in the *CDKN2A* locus are associated with differential expression of P16INK4A and P14ARF in HPV-positive oropharyngeal squamous cell carcinoma

**DOI:** 10.1002/cam4.374

**Published:** 2015-01-26

**Authors:** Nicolas F Schlecht, Miriam Ben-Dayan, Nicole Anayannis, Roberto A Lleras, Carlos Thomas, Yanhua Wang, Richard V Smith, Robert D Burk, Thomas M Harris, Geoffrey Childs, Thomas J Ow, Michael B Prystowsky, Thomas J Belbin

**Affiliations:** 1 Department of Epidemiology & Population Health, Albert Einstein College of Medicine1300 Morris Park Avenue, Bronx, New York, 10461; 2 Department of Medicine (Oncology), Albert Einstein College of Medicine1300 Morris Park Avenue, Bronx, New York, 10461; 3 Department of Pathology, Albert Einstein College of Medicine1300 Morris Park Avenue, Bronx, New York, 10461; 4 Department of Otorhinolaryngology-Head and Neck Surgery, Montefiore Medical CenterMedical Arts Pavilion, 3400 Bainbridge Avenue, Bronx, New York, 10467; 5 Department of Pediatrics (Genetics), Microbiology & Immunology; Obstetrics, Gynecology & Women's Health, Albert Einstein College of Medicine1300 Morris Park Avenue, Bronx, New York, 10461

**Keywords:** Carcinoma, HPV, methylation, oropharynx, squamous

## Abstract

Human papillomavirus (HPV)-positive oropharyngeal squamous cell carcinoma (OPSCC) is recognized as a distinct disease entity associated with improved survival. DNA hypermethylation profiles differ significantly by HPV status suggesting that a specific subset of methylated CpG loci could give mechanistic insight into HPV-driven OPSCC. We analyzed genome-wide DNA methylation of primary tumor samples and adjacent normal mucosa from 46 OPSCC patients undergoing treatment at Montefiore Medical Center, Bronx, NY using the Illumina HumanMethylation27 beadchip. For each matched tissue set, we measured differentially methylated CpG loci using a change in methylation level (M value). From these analyses, we identified a 22 CpG loci panel for HPV+ OPSCC that included four *CDKN2A* loci downstream of the p16(INK4A) and p14(ARF) transcription start sites. This panel was significantly associated with overall HPV detection (*P* < 0.05; ROC area under the curve = 0.96, 95% CI: 0.91–1.0) similar to the subset of four *CDKN2A*-specific CpG loci (0.90, 95% CI: 0.82–0.99) with equivalence to the full 22 CpG panel. DNA hypermethylation correlated with a significant increase in alternative open reading frame (ARF) expression in HPV+ OPSCC primary tumors, but not to the other transcript variant encoded by the *CDKN2A* locus. Overall, this study provides evidence of epigenetic changes to the downstream region of the *CDKN2A* locus in HPV+ oropharyngeal cancer that are associated with changes in expression of the coded protein products.

## Introduction

Human papillomavirus (HPV)-positive oropharyngeal squamous cell carcinoma (OPSCC) is recognized as a distinct disease associated with improved survival and response to therapy, and carries distinct pathologic and molecular characteristics, including positive immunostaining for the p16 protein 1. Results from molecular array studies also support the hypothesis that HPV-positive and negative OPSCC are biologically distinct and may represent different cancer lineages formed through separate etiologic pathways of multistage tumorigenesis 2,3. Despite the recognized importance of HPV in OPSCC, the pathobiology of HPV in OPSCC tumors is not well understood.

HPV detected in OPSCC tumors (HPV16, the most common high-risk type also found in cervical cancer) is frequently integrated and transcriptionally active, with the majority of HPV+ tumors expressing the viral oncoproteins E6 and E7 4. HPV oncoproteins E6 and E7 interact with and inactivate the cellular tumor suppressor proteins p53 and pRb, respectively, and are responsible for disrupting the p16(INK4A) tumor suppressor pathway 5. P16 protein expression is associated with improved survival among patients with OPSCC 6, and p16 immunostaining is used routinely in a clinical setting as a surrogate marker of HPV infection in OPSCC, but shows poor clinical utility in nonoropharyngeal head and neck cancers 7,8. The *CDKN2A* gene, which codes for both the p16 protein and its alternate reading frame (p14ARF) is frequently affected by epigenetic gene silencing and deletion in other head and neck cancers, and both have been established as known tumor suppressors in head and neck carcinogenesis 9. In spite of their structural and functional differences, both proteins share a common functionality in cell cycle control. This ARF product functions as a stabilizer of the tumor suppressor protein p53 via its interaction and inhibition of the E3 ubiquitin-protein ligase MDM2. Its partner p16 is an inhibitor of cyclin-dependent kinases such as CDK4 and CDK6.

As in other cancers, multiple genetic and epigenetic events have been implicated in head and neck carcinogenesis 10. Our group has recently shown a significantly higher number of differentially methylated CpG loci in HPV+ OPSCC tumors compared to their HPV− counterparts 11. We now identify a novel 22 CpG loci panel that is significantly associated with HPV detection and p16 expression in OPSCC patients from our population as well as in an independent cohort of OPSCC from the Cancer Genome Atlas (TCGA) project. Included in this panel are four CpG loci located downstream of the p16(INK4A) transcription start site that are hypermethylated in HPV+ OPSCC primary tumors compared to normal tissues, and associated with increased expression of the p14(ARF) gene product. Our results establish a potential mechanism connecting HPV positivity and expression of *CDKN2A* transcripts that is separate from tumor suppressor proteins p53 and pRb, and may involve direct epigenetic regulation of ARF transcription.

## Methods

### Study population

All patients consented to participation in this study under protocols approved by the Institutional Review Boards of the participating institutions. All primary tumor and histologically normal adjacent tissues were snap-frozen in liquid nitrogen within 30 min of surgical resection or biopsy and kept at −80°C until further processing. Adjacent mucosa was taken at a grossly unremarkable site away from the tumor identified by the surgeon (incisional biopsy) intraoperatively or by the pathologist (resection specimen) when processing the specimen for frozen section or final diagnosis as described previously 11. TNM staging was based on the AJCC classification. Control samples for all tumors were acquired; microscopic slides stained with hematoxylin and eosin were assessed for percentage of tumor, extent of necrosis, and degree of lymphocytic infiltration as described previously 11.

### HPV testing of patient samples

All patient samples were tested for HPV infection using multiple testing methods. The presence of HPV DNA was assayed by MY09/11-PCR (degenerate and type-specific HPV16-L1 fragments) utilizing AmpliTaq Gold (Applied Biosystems Life Technologies, Grand Island, NY, USA) and dot-blot hybridization on fresh frozen specimens as described previously 7. The integrity of specimen DNA was verified by amplification of a fragment of the *β*-globin gene. In addition, HPV-positive and negative control samples were included in each PCR using DNA from a cervical carcinoma cell line (SiHa) with two copies of HPV16 per cell and a HPV-negative cell line HepG2.

We also tested for HPV16 RNA expression by reverse transcription PCR (RT-PCR). RNA extractions were tested for HPV16 transcripts using oligonucleotide primers that span the 204–525 base-pair regions of the *E6* and *E7* oncogenes 12. All gene expression was normalized to the control probe glyceraldehyde-3-phosphate dehydrogenase (*GAPDH*) as an RNA control (sequence AY340484, complete cds). Assays were performed in triplicate on the StepOnePlus™ Real-Time PCR system. Only patients testing positive by all PCR methods were classified as HPV positive (Table1), while those testing negative by both were classified as negative. Those not confirmed by both methods were considered indeterminate.

**Table 1:** Clinical characteristics of oropharyngeal cancer patients

	Einstein cohort (*N* = 46)		TCGA cohort (*N* = 33)	
	*N*	%	*N*	%
Gender				
Male	37	80	27	82
Female	9	20	6	18
Median age (range)	62 (40–84)		56 (35–79)	
Race				
White	27	58	30	91
African–American	18	38	3	9
Asian	0	0		
Unknown	1	2		
Ethnicity				
Hispanic/Latino	9	20	1	3
Non-Hispanic/Latino	36	78	31	94
Unknown	1	2	1	3
Smoking				
Current	19	41		
Former	23	50		
Never	4	9		
Tumor stage				
I	7	15	3	0
II	5	10	4	32
III	4	8	3	10
IV	30	65	7	45
Unknown			16	49
Node status				
N0	14	31	8	24
N1	4	8	1	3
N2	23	50	5	15
N3	5	11	1	3
Unknown	0	0	18	55
HPV status				
HPV (+)	15	33	21	64
HPV (-)	23	50	11	33
Indeterminate	8	17	1	3
P16 status				
P16 (+)	16	35		
P16 (-)	25	54		
Unknown	5	11		

### Whole-genome DNA methylation

Genome-wide profiling of DNA methylation in oropharyngeal primary tumor samples was carried out as described previously 11. Normalized M values were generated from primary HumanMethylation27 beadchip beta values using the R package HumMeth27K QC Report function, including the X chromosome data, and using an average probe *P*-value of 0.03 as the cutoff for sample inclusion 13. A total of 25,593 CpG loci satisfied these criteria for all samples. Simultaneous whole-genome expression analysis was carried out for all Einstein oropharyngeal tumor samples by hybridization of amplified RNA to an Illumina HumanHT-12 v3 Expression BeadChip according to the directions of the manufacturer (Illumina Inc.; San Diego, CA). All expression data were quantile normalized and background-subtracted prior to analysis using BeadStudio software (Illumina Inc.).

### *CDKN2A*(p16) protein and mRNA expression

Initial measurements of p16 protein positivity in OPSCC tumors were assayed using standard immunohistochemical techniques. All tissues were routinely fixed in 10% buffered formalin and embedded in paraffin. The paraffin sections were cut at 4-*μ*m thickness and were placed on the positively charged slides. Slides were placed in a 60°C oven for an hour. Sections were deparaffinized and rehydrated through a series of xylene and graded alcohols (100%, 95%, and 75%). Endogenous peroxidase was blocked in 3% H_2_O_2_ for 10 min. Antigen retrieval was performed by placing the slides in an Oyster vegetable steamer with Dako Target Retrieval Solution (S1699; Dako North America, Inc.; Carpinteria, CA). The immunoperoxidase staining procedure was performed in an automatic slide stainer (Dako Autostainer Plus) using the Dako universal staining system. The primary mouse monoclonal antibody p16 (551154; BD Biosciences; San Jose, CA) was applied in a dilution of 1:50 for 30 min at room temperature. After primary antibody, the slides are washed buffered solution, a secondary antibody (Dako Envision+ system-HRP Labeled Polymer, antimouse; K400111) was applied for 30 min. DAB Substrate kit (Dako; K346811) was used with 3,3′-diaminobenzidine as chromogen. Slides were counterstained with Surgipath Hematoxylin (01560; Leica Microsystems; Buffalo Grove, IL), dehydrated through graded alcohols, cleared in xylene, and cover-slipped with cytoseal 60 (8310; Richard-Allan Scientific; Kalamazoo, MI).

Subsequently, measurements of individual *CDKN2A* mRNA splice variants in oropharyngeal tumors and normal tissues were carried out using the real-time quantitative PCR measurements (qRT-PCR) with the Applied Biosystems® (Applied Biosystems Life Technologies, Grand Island, NY, USA) TaqMan® RNA-to-Ct™ 1-Step Kit. Four primers were used to distinguish the mRNA transcripts: Hs00924091 (p14 ARF), Hs00923893 (p12), Hs02902543 (p16 gamma and INK4A), and Hs00923894 (p14 ARF, p16 INK4A, and p12). Sample cycle threshold (C_t_) values were normalized to the *GAPDH* control, and the samples with no mRNA detection were set as the baseline. Relative C_t_ values were converted to a relative expression (log_2_-C_t_). To graph the expression ratios, the log_2_(tumor/normal C_t_s) was used for each tumor:normal pair. To graph the HPV+ tumor samples only, the relative expression (log_2_) was used. All samples were examined in triplicates and the mean value taken.

### Measurement of *CDKN2A* CpG loci DNA methylation by bisulfite sequencing

Validation of the *CDKN2A* beadchip measurements was carried out by bisulfite sequencing. Genomic DNA was bisulfite converted as described above. Two regions located within a downstream CpG island that is shared by ARF and INK4A were targeted using this approach. Primer sequences, PCR conditions, and amplicon sizes are shown in Data S1. The targeted PCR products were amplified using the Amplitaq Gold 360 Polymerase Kit from Applied Biosystems and sequenced by Albert Einstein College of Medicine Genomics Core.

### Statistical analyses and validation

For each tumor:normal tissue pair, we calculated Δ*M* (*M*_tumor_−*M*_normal_) for the 25,593 CpG loci on the beadchip that passed initial quality control (average probe *P*-value <0.03 for all samples). To identify a training sample set, we overlayed HPV data, based on both DNA and E6 mRNA PCR results, with p16 protein tumor expression by immunohistochemistry. Samples showing concordant results were selected including 13 OPSCC with strong HPV DNA and RNA positivity and p16 protein staining, and 10 HPV−, p16- OPSCC. P16 protein immunohistochemistry results were initially missing for 10 OPSCC cases included in our previous analyses previously 11. We then determined statistical significance of the differences in ΔM for each CpG loci between the two sample populations (HPV+/p16+ vs. HPV−/p16-) using Wilcoxon rank sum test with Bonferroni correction. CpG loci were ranked based on the *P*-value as well as the magnitude of difference in methylation changes between the two groups. From the training set, we identified 23 CpG loci with a *P*-value of less than 1 × 10^−4^, of which 22 showed at least a 1.5-fold difference in average ΔM between HPV+/p16+ and HPV−/p16− cases. A heatmap for these 22 highest ranked CpG loci was then generated using the Cluster program with median centering of CpG loci, hierarchical clustering, and Euclidian distance 14.

Differences in methylation and expression between HPV+ and HPV− cases were assessed using parametric and nonparametric univariate tests. Nonparametric receiver operating characteristic (ROC) plots reflecting combined test sensitivity and specificity were generated to illustrate the degree of association with HPV and p16 status in OPSCC, including an additional 22 cases not tested in the training set. Cumulative methylation scores were generated based on tumor-to-normal methylation (ΔM) levels across the 22 CpG panel and separately for the four *CDKN2A* loci downstream of the p16(INK4A) and p14(ARF) transcription start sites.

### Validation of DNA methylation and gene expression results using the Cancer Genome Atlas

We validated the performance of the methylation panel in an external cohort of 33 HPV+ and HPV− OPSCC cases from TCGA project (https://tcga-data.nci.nih.gov/tcga) (Table1). Tumor methylation data (M values) for CpG loci corresponding to the 22 CpG loci were extracted from DNA methylation data sets corresponding to these patients, and the samples sorted using hierarchical clustering. RNA-Seq data corresponding to the 33 OPSCC tumors from the TCGA were extracted for p14(ARF) and p16(INK4A) (https://tcga-data.nci.nih.gov/tcga/tcgaCancerDetails.jsp?diseaseType=HNSC&diseaseName=Head and Neck squamous cell carcinoma). Methods for sequencing and data processing of RNA using the RNA-Seq protocol have been previously described for TCGA 15. Briefly, RNA-Seq data were generated using Illumina TruSeq mRNA libraries, and sequenced by Illumina HiSeq2000. Workflow for processing and normalization of these data is described elsewhere (https://cghub.ucsc.edu/docs/tcga/UNC_mRNAseq_summary.pdf). Expression results were presented as normalized read counts. DNA methylation and gene expression data were not normalized or processed by our group in any way prior to analysis.

## Results

### HPV-positive OPSCC tumors display a unique epigenetic profile

**Figure 1:**
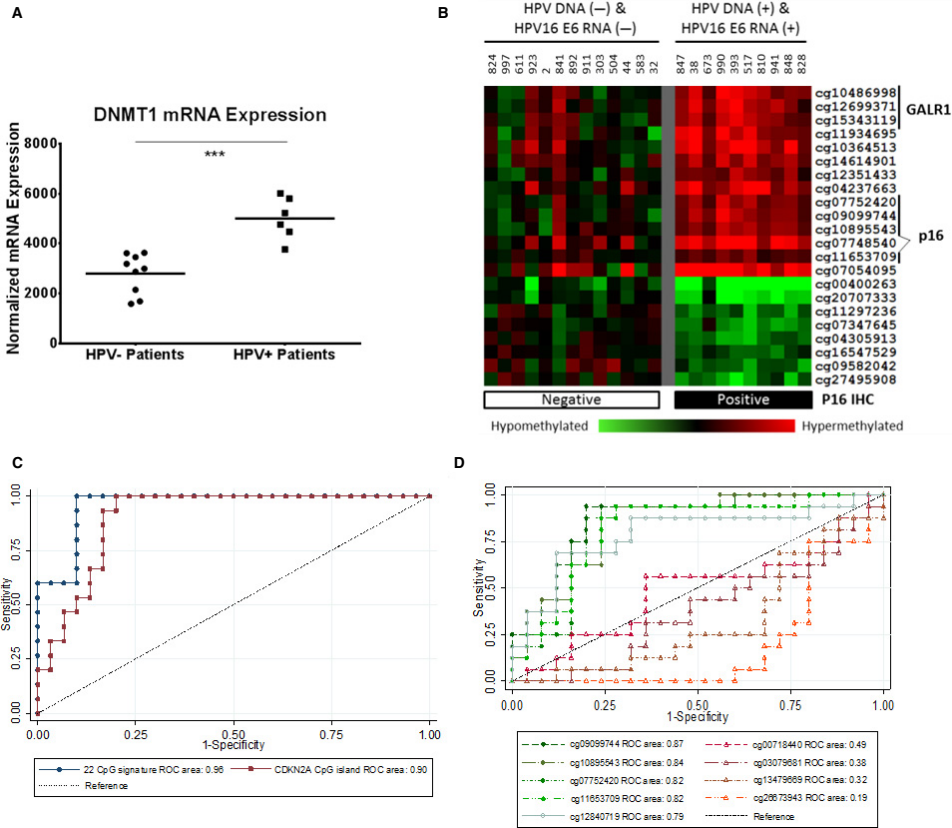
(A) Dot plot of normalized DNMT1 expression, as determined by beadchip probe fluorescence intensity (ILMN_1760201 probe) in total RNA samples of 9 HPV-negative and 6 HPV-positive OPSCC primary tumors obtained previously from the Albert Einstein head and neck cancer database. Statistical significance was calculated by unpaired *t*-test with Welsch correction (***P < 0.001). (B) Heat Map of the 22 CpG molecular panel distinguishing HPV-positive from HPV-negative OPSCC. Anonymized patient identifiers are included for the 23 OPSCC patients for which HPV and p16 IHC status were available (10 HPV−p16-, 13 HPV+p16+). Presence of HPV16 DNA and RNA (top) and p16 immunohistochemistry (bottom) status for each patient are shown. Illumina TargetID for each CpG loci is indicated at the right. DNA hypermethylation (tumor to corresponding normal) is shown in red; DNA hypomethylation is indicated by green. CpG loci associated with the GALR1 and p16 genes are indicated at the right. A self-organizing map (SOM) was used to cluster CpG loci by methylation status using Cluster and the results were visualized with TreeView. P16 immunopositivity was assayed using standard immunohistochemical techniques. (C) Receiver operating characteristic (ROC) plots against tumor HPV positivity in an expanded set of 46 OPSCC defined based on a combination of HPV-DNA and RNA detection by PCR and tumor p16 protein expression by immunohistochemistry (see Results section for details). Using tumor-to-normal ΔM values for the 22 CpG molecular panel (•) and separately using four CpG loci within the downstream region of the CDKN2A (▪), ROC areas under the curve for both panels were significantly better at predicting HPV status than chance at *P* < 0.05. (D) ROC plots predicting p16 positivity in 46 OPSCC by immunohistochemistry. Tumor-to-normal (ΔM) values for nine CpG sites on the Illumina HumanMethylation27 BeadChip, including five located in the CpG island (9:21958106-21958899) downstream of the transcriptional start site (cg11653709, cg12840719, cg09099744, cg07752420, cg10895543) (○) and four within the CDKN2A promoter (-Δ-) (9:21983444-21986348) (cg03079681, cg13479669, cg26673943, and cg00718440).

We showed previously that HPV+ oropharyngeal cancers exhibit significantly higher numbers of differentially methylated CpG loci (hyper- and hypomethylated) between tumors and adjacent normal mucosa compared to HPV− oropharyngeal, oral cavity, and laryngeal cancers 11 suggesting that a specific subset of CpG loci may be identified that could further stratify these cancers and reflect DNA methylation changes associated with HPV. We tested a total of 46 patients undergoing treatment for histologically confirmed OPSCC at Montefiore Medical Center, Bronx, NY (Table1). When mining gene expression data from our head and neck cancer genomics database, HPV+ OPSCC tumors showed a significant increase in the expression of DNA methyltransferase 1 (*DNMT1*), the major eukaryotic DNA methyltranferase (unpaired *t*-test with Welsch correction, *P* < 0.001) (Fig.1A) supporting our observation of increased DNA methylation in these tumors 11.

By analyzing genome-wide DNA methylation between tumor and matched normal tissue samples for a subset of OPSCC patients, 22 CpG loci were identified that showed a statistically significant difference in methylation (ΔM) when comparing HPV+ to HPV− OPSCC (Fig.1B). Among these 22 CpG loci were four *CDKN2A*-specific loci located downstream of the p16(INK4A) transcription start site, two loci located within the CpG island of the *GALR1* gene, and one associated with the *PPP1R3D* gene.

### DNA methylation of the 22 CpG, including the 4 CpG *CDKN2A*-specific loci, is associated with HPV detection and p16 protein expression in OPSCC

We then extended our analyses to an additional 22 OPSCC patients, including: 9 new HPV−/p16- tumors plus 8 ambiguous cases (1 HPV DNA-/p16+ tumor and 7 tumors with indeterminate or low levels of HPV DNA and RNA), and 4 HPV16 DNA/RNA++/p16+ tumors plus 1 case positive for a related high-risk type (HPV35) and p16. We observed significant differences in overall DNA methylation levels between HPV+ and HPV− OPSCC cases for the 22 CpG panel (Wilcoxon rank sum test *P* < 0.00005), which extended to both subsets of CpG sites within the panel that showed either hypermethylation (*P* < 0.00005) or hypomethylation (*P* < 0.00005) in HPV+ versus HPV− OPSCC cases. We also observed significant correlations between DNA methylation levels and HPV16 oncogene expression levels measured by qRT-PCR for both the E6 (Spearman *ρ* = 0.54, *P* = 0.0016) and E7 oncogenes (*ρ* = 0.47, *P* = 0.007).

We generated ROC plots to illustrate the degree of association with overall HPV detection in OPSCC, including an additional 22 cases not tested in the training set as well as the HPV35+ case (Fig.1C). As reflected by the ROC area under the curve (AUC), the combined methylation across the 22 CpGs was significantly associated with overall HPV positivity (AUC = 0.96, 95% confidence interval [CI]: 0.91–1.0). Concordance based just on the subset of four downstream *CDKN2A* CpGs (cg11653709, cg09099744, cg07752420, cg10895543) was also significant (AUC = 0.90, 95% CI: 0.82-0.99) and statistically equivalent to the full 22 CpG signature (*P* = 0.084).

The Illumina HumanMethylation27 BeadChip contains nine CpG sites associated with the *CDKN2A* gene: five in the CpG island (9:21958106-21958899) located downstream of the p16(INK4A) transcription start site (cg11653709, cg12840719, cg09099744, cg07752420, cg10895543), of which four were identified in our HPV panel, and another four located within the CpG island of the *CDKN2A* promoter region (9:21983444-21986348; cg03079681, cg13479669, cg26673943, and cg00718440). We compared the tumor-to-normal methylation (ΔM) ratios for each of the nine individual loci to p16 protein expression on histology using ROC analyses (Fig.1D). CpG sites located downstream of the p16(INK4A) transcription start site predicted p16 positivity (*P* < 0.05) with AUC ranging from 0.79 to 0.87. In contrast, those located within the *CDKN2A* promoter region showed no association or an inverse association with AUC ranging from 0.49 to 0.19.

### DNA methylation of the *CDKN2A* locus is correlated with increased tumor expression of p14(ARF) in OPSCC

**Figure 2:**
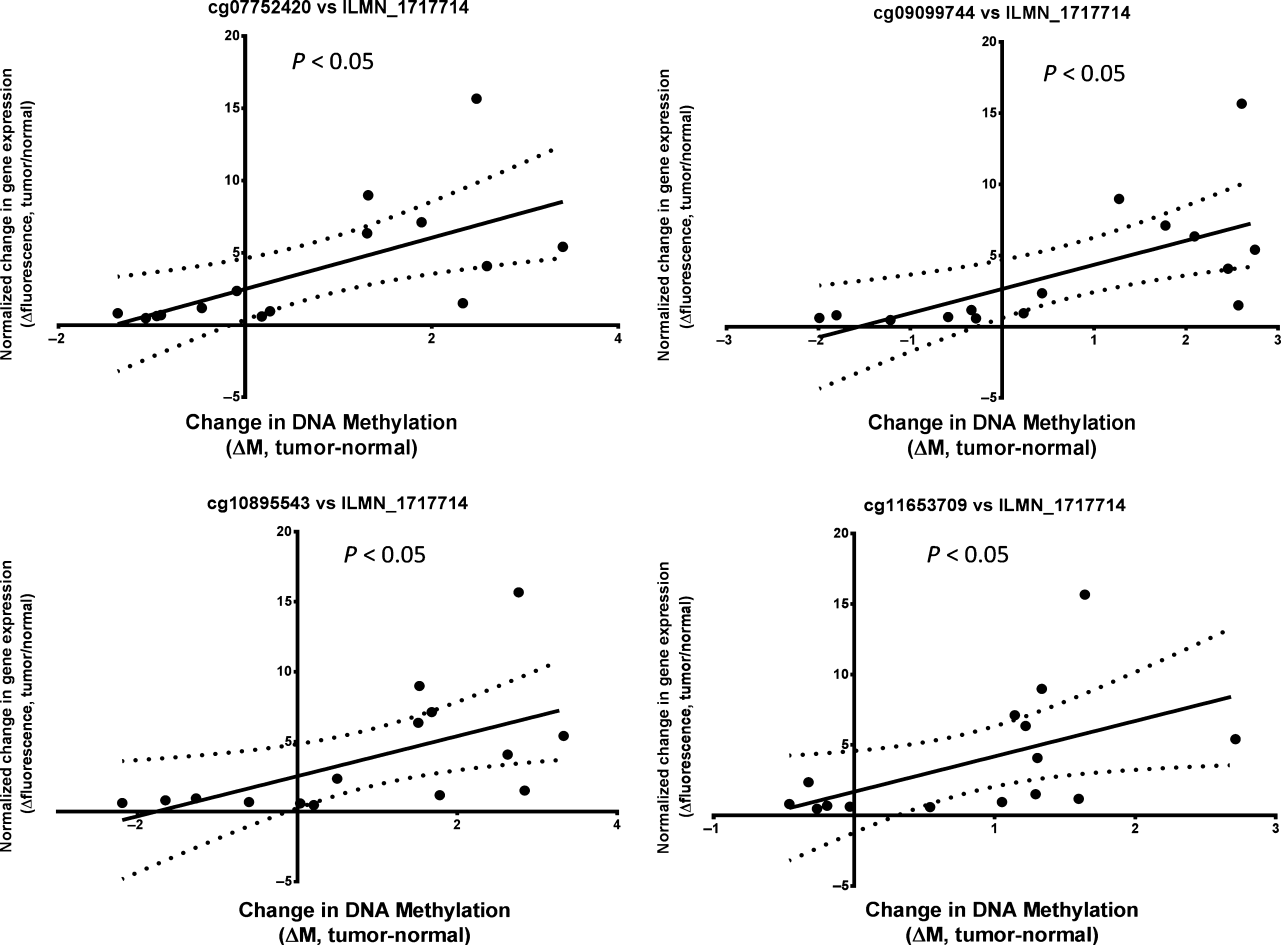
Scatter plot of normalized change in gene expression, as measured by beadchip probe fluorescence intensity (ILMN_1717714) versus a corresponding change in DNA methylation (ΔM, tumor-normal) for 15 HPV-negative and HPV-positive OPSCC primary tumors. Tumor/normal pairs were selected from the 46 OPSCC patients which had overlapping gene expression (Illumina HT-12 expression beadchip) data for both tissue types. Four separate CpG loci (cg11653709, cg09099744, cg07752420, cg10895543), located downstream of the start site for the CDKN2A gene, are shown. Statistical significance was calculated by linear regression. Dotted lines indicate 95% confidence intervals.

Many genes represented in the 22 CpG panel also showed differences in gene expression as measured by the Illumina HumanHT-12 v3 Expression BeadChip (Illumina Inc.) comparing beadchip probe fluorescence intensities for tumor and normal tissue mRNA samples collected on the same OPSCC patients, including *CDKN2A* (expression probe ILMN_1717714), *GPSM1* (ILMN_1709307), *FLJ33790/KLHL35* (ILMN_1693471), and *SYCP2* (probes ILMN_2095704 and ILMN_1765770) 11. For the CpG sites identified in our panel for *CDKN2A*, *KLHL35* and *SYCP2*, there were significant positive correlations between observed levels of DNA methylation and expression of the corresponding genes. In particular, methylation of the four CpG loci located in the CpG island (9:21958106-21958899) downstream of the p16(INK4A) and p14(ARF) transcriptional start sites (cg07752420, cg09099744, cg10895543, and cg11653709), were significantly correlated with expression of the *CDKN2A* gene as measured by probe ILMN_1717714 (Fig.2). It was somewhat surprising that this correlation between DNA methylation and mRNA expression was opposite to what would be expected for an epigenetically silenced gene. It is possible that these four CpG loci (positions chr9: 21958681, 21958372, 21958832, and 21958146), may reveal a novel epigenetic mechanism, whereby increased DNA methylation at a downstream regulatory site acts to increase expression of mRNA transcripts originating from the *CDKN2A* locus.

**Figure 3:**
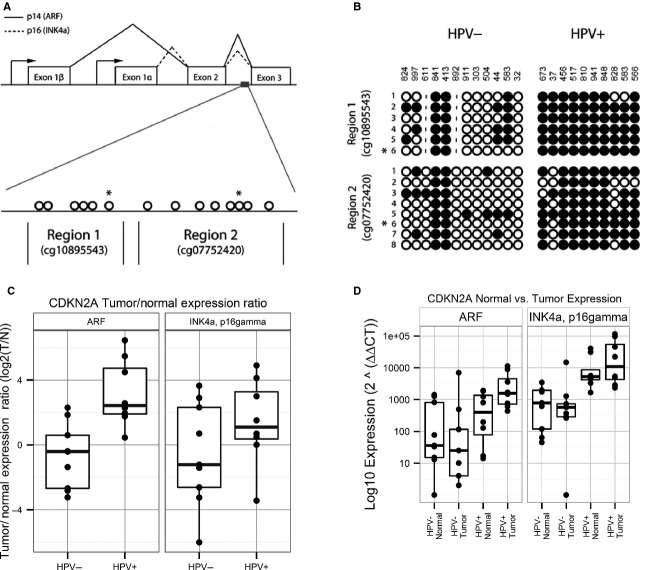
(A) Genomic organization of the chromosome 9p21 CDKN2A locus encoding p16^INK4A^ and p14^ARF^. Proteins p16(INK4A) and p14(ARF) share exons 2 and 3 but have a distinct exon 1. RNA transcripts are translated in different reading frames to generate two separate protein products. Alternate promoter sites are indicated by arrows. Location of the CpG island corresponding to the downstream region (9:21958106-21958899) is indicated by a grey box. Also shown are two DNA segments located within the CDKN2A downstream region that was tested for DNA methylation by bisulfite sequencing. Individual CpG loci to be tested within each region are indicated by open circles; those indicated with an asterisk correspond to the location of Illumina beadchip loci cg10895543 and cg07752420. (B) Measurement of CpG methylation observed in the *CDKN2A* downstream region of HPV+ and HPV− primary OPSCC tumors by bisulfite sequencing. Patient identifiers are indicated on the top; individual CpG loci for each region are shown at the left. Methylated CpG loci are indicated by closed circles; unmethylated CpG loci are indicated by open circles. Positions of CpG loci included on the Illumina HumanMethylation27 BeadChip are indicated by an asterisk. (C) Real-time PCR measurements of gene expression for p14(ARF), p16(INK4A), and p16 gamma in oropharyngeal tumor and adjacent normal tissues from HPV-positive and HPV-negative oropharyngeal cancer cases. The panel shows expression changes (tumor/normal ratio) in 8 HPV+ and HPV− tumor samples following log_2_ transformation. Statistical significance of gene expression differences between HPV-positive and HPV-negative cases were assessed by Wilcoxon Rank Sum. (D) Analysis of p14(ARF), p16(INK4A), and p16 gamma expression looking individually at normal adjacent mucosa and primary OPSCC tumors as independent populations in HPV-positive and HPV-negative patients.

It is known that the *CDKN2A* region is highly complex, coding for mRNA transcripts corresponding to multiple splice variants, including p14(ARF), p12, p16(gamma), and p16(INK4A) (Fig.3A). We evaluated whether differential methylation was associated with transcription of these splice variants. First, we validated differential DNA methylation of the affected downstream *CDKN2A* region by bisulfite sequencing. Two subregions were sequenced, each including a CpG locus from the original panel (Region 1: cg10895543, Region 2: cg07752420), as well as five to seven additional CpG loci located within the same subregions. A total of 17 OPSCC tumor samples from the training set (used to develop the methylation panel) and 5 OPSCC tumors from the test set were sequenced. For both subregions, bisulfite sequencing revealed an increase in DNA methylation in HPV+ compared to HPV− OPSCC tumors (Fig.3B). This pattern was observed for the overlapping CpG loci identified from the Illumina panel (cg10895543 and cg07752420), as well as for the surrounding adjacent CpG loci.

In order to examine which transcript variants were specifically affected by DNA methylation in *CDKN2A*, RNA expression of p16(INK4A), p16(gamma), p14(ARF), and p12 splice variants in OPSCC tumors and adjacent normal tissues were measured by qRT-PCR (Fig.3C). Although there was no significant change in p16(INK4A) expression ratios (primary tumor compared to corresponding adjacent mucosa from the same patient) between HPV− and HPV+ OPSCC (Wilcoxon rank sum test *P* = 0.3), we observed a significant increase in p14(ARF) expression (tumor:normal ratio) in HPV+ compared to HPV− OPSCC (*P* = 0.004).

Since these differences in the expression ratios could be attributed to changes in either tumor expression, normal tissue expression, or both, we repeated the analysis of gene expression looking at these tissues separately (Fig.3D). When analyzed separately, we observed an increase in expression of p16(INK4A) in both tumor and adjacent normal tissues from HPV+ OPSCC compared to HPV− OPSCC (Wilcoxon rank sum test *P* = 0.007 and *P* = 0.006, respectively), but little difference in overall expression of p16(INK4A) between tumor and adjacent normal samples within the same subject for either HPV+ or HPV− OPSCC cases. In contrast, we observed a significant increase in p14(ARF) expression in HPV+ tumors compared to HPV− tumors (*P* = 0.001), whereas p14(ARF) expression levels in the adjacent normal tissues from HPV+ and HPV− cases were not statistically significant different (*P* = 0.3). Therefore, the changes in DNA methylation observed within the downstream CpG island of the *CDKN2A* locus are correlated with a tumor-specific increase in p14(ARF) mRNA expression. Further studies are required to detail the mechanism by which the downstream region regulates the expression of these two mRNA transcripts.

### Differences in DNA methylation of *CDKN2A*-specific CpG loci and expression of p14(ARF) and p16(INK4A) are associated with HPV detection in OPSCC tumors from TCGA

**Figure 4:**
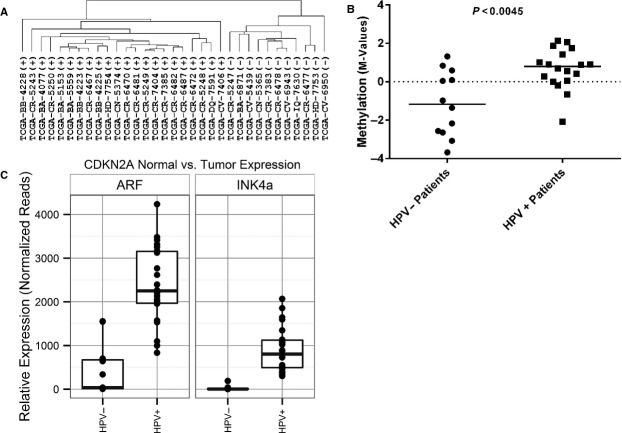
(A) Hierarchical clustering of 33 oropharyngeal cancer cases from the Cancer Genome Atlas (TCGA) based on the methylation status of the 22 CpG loci. HPV status of individual TCGA cases (+ or −) is shown. (B) Measurements of DNA methylation (M-value) for *CDKN2A* downstream locus cg12840719 in 30 oropharyngeal primary tumors (12 HPV-, 18 HPV+) obtained directly from the Cancer Genome Atlas (TCGA). (C) Expression of ARF and INK4A in the same 33 oropharyngeal cancer cases from the Cancer Genome Atlas (TCGA) based on publically available RNA-Seq data. Expression of ARF and INK4A are plotted as normalized read counts based on processing of the data as described previously by the TCGA analysis team^15^.

Given the limited availability of DNA methylation data related to normal adjacent mucosa from TCGA, we applied our methylation panel to primary OPSCC tumor samples alone with known HPV status (11 HPV−, 22 HPV+). Hierarchical clustering using tumor DNA methylation levels revealed two clusters that segregated samples by HPV status (Fig.4A). Since the four CpG loci associated with *CDKN2A* were not present in the Illumina platform used to generate TCGA data sets, we confirmed the association between methylation of the downstream region of *CDKN2A* and HPV status by using an alternate CpG locus in the same *CDKN2A* downstream region not present in our panel but found in the TCGA data set (Table1). With this data, we found that DNA methylation at this *CDKN2A* downstream locus (cg12840719) showed significantly higher methylation levels (M values) in HPV+ compared to HPV− OPSCC cases from TCGA (Wilcoxon rank sum test *P* = 0.0045; Fig.4B). Similarly, significant increases were seen in mRNA expression of p14(ARF) and p16(INK4A) among HPV+ compared to HPV− OPSCC from the TCGA (Fig.4C). Median p14(ARF) expression counts increased from 442 in HPV− cases to 2392 in HPV+ cases (Wilcoxon rank sum test *P* < 0.001), whereas median p16(INK4) expression counts increased from 23 in HPV− cases to 906 in HPV+ cases (*P* < 0.001). In summary, we observed that differential DNA methylation of CpG loci from the same regions identified in our novel panel, and in particular the *CDKN2A*-specific downstream region, was significantly associated with HPV detection in OPSCC in two independent cohorts. Moreover, these changes correlated with an increase in expression of the *CDKN2A* splice variant p14(ARF).

## Discussion

The experiments described here assess DNA methylation and gene expression difference in OPSCC patients characterized by HPV DNA and RNA detection and p16 protein expression. Our previous work using the Illumina HumanMethylation27 beadchip established a significant increase in DNA methylation in HPV+ oropharyngeal cases as compared to HPV− cases 11. The results presented here reveal a corresponding increase in expression of *DNMT1*, the major mammalian DNA methyltransferase, in HPV+ tumors. Further analysis identified a subset of 22 CpG that were significantly associated with detection of HPV in OPSCC.

Other studies have shown that HPV+ OPSCC tumors have higher levels of gene promoter methylation compared with HPV− tumors 16. Kostareli et al. 17 utilized an array-based approach to identify specific alterations in genome-wide promoter methylation in HPV+ OPSCCs and identified a signature that predicted clinical outcome. Similarly, Lechner et al. 18 identified 43 hypermethylated promoter regions associated with HPV in head and neck cancers, including three cadherins of the Polycomb group target genes. Curiously, these methylation-based panels shared little, if any, common elements. This may be due to the large number of methylation differences between HPV+ and HPV− head and neck tumors and small sample sizes used in the two studies, or to the different experimental platforms employed in these studies. The HPV panel described here is the first to include epigenetic changes associated with the *CDKN2A* locus. One of the *CDKN2A* products (the p16^INK4A^ protein) is commonly used as a clinical diagnostic, and has been advocated as a surrogate marker for HPV in OPSCC 1. Aberrant methylation events, including some associated with *CDKN2A*, have been observed in head and neck cancer cell lines, as well as in cervical cancers 19–21. Interestingly, previous studies have also shown that the *CDKN2A* locus, which is normally repressed in cycling cells by *EZH2* via *H3K27* methylation*,* and frequently undergoes DNA hypermethylation in cancer, is often overexpressed in HPV+ carcinoma 22. Examination of the DNA methylation of the 22 CpG loci identified here, particularly those associated with the *CDKN2A* region, within nonoropharyngeal head and neck cancer cases is ongoing.

It is known that there are both protein mediated and epigenetic mechanisms that regulate the transcription of p14(ARF). For example, the HPV16 oncoprotein E7 causes the release of E2F-1 from its repressor Rb 23, while the E2F-1 transcription factor promotes the transcription of important S phase entry proteins and ARF 24. There is also regulatory feedback loop between ARF and p53, where p53 functions as an p14(ARF) transcriptional repressor and ARF prevents p53 degradation 25. HPV16 oncoprotein E6 promotes the degradation of p53, thereby removing its inhibitory effects on p14(ARF) transcription 26.

Overall, the results implicate hypermethylation of these downstream CpG sites in the *CDKN2A* gene as a potential mechanism for increased p16 expression in HPV+ OPSCC tumors. However, the mechanism by which the hypermethylated downstream CpG island region leads to altered expression of transcripts originating from the *CDKN2A* locus is not clear. Our assessment of the region reveals a large number of Sp1 binding sites, a pattern observed previously in downstream CpG island regions involved in transcription initiation, particularly those of some long noncoding RNAs 27. It has also been demonstrated that a specific Sp1 recognition sequence is critical for the transactivation of the *TGF-β1* promoter by HPV16 E6 and E7 in cervical cancer 28. Taken together, the epigenetic mechanisms described above, combined with our description of a potential downstream regulatory region demonstrating DNA hypermethylation in OPSCC tumors, provide a plausible hypothesis that HPV may induce an increase in expression of *CDKN2A* transcripts (specifically p14(ARF)) in OPSCC.

A number of limitations should be considered when interpreting these results. While the 22 CpG loci panel best distinguished HPV infection in this cohort, we are cognizant of the fact that host genetic events such as mutations observed in p53 and p16 (possibly caused by tobacco carcinogens) may confound associations between HPV infection, panel DNA methylation, p16 expression, and clinical outcome. The specific genotyping of this population as it relates to those events will be the focus of future work by our group. While the agreement in associations seen with TCGA supports our findings, it should be noted that our study sample was small and represented a subset of cases from a larger cancer patient cohort; its probable additional methylation changes may better resolve ambiguous primary tumors for which there is discordance between HPV assays. It should also be pointed out that our study was focused specifically on host genome DNA methylation. DNA methylation of the HPV viral genome will be the focus of a future study. And finally, it is not clear whether viral proteins are directly involved in the methylation of the CpG loci downstream of the CDKN2A gene promoter, or whether this relies on other ancillary proteins.

In summary, we observed that HPV-positive OPSCC has significantly increased *DNMT1* expression compared to HPV− cases. Further whole-genome DNA methylation analysis identified a subset of 22 CpG that were significantly associated with HPV detection in OPSCC in two independent cohorts. Within this panel, we identified a hypermethylated region downstream of the *CDKN2A* gene that was associated with p16 protein expression in OPSCC and is correlated with an increase in tumor-specific expression of the *CDKN2A* transcript variant p14(ARF).

## Supporting Information

**Data S1:** Bisulfite PCR sequencing primers and conditions.
