# Supplementary material for: Epigenetic changes in the CDKN2A locus are associated with differential expression of P16INK4A and P14ARF in HPV-positive oropharyngeal squamous cell carcinoma
Source: Cancer Med. 2015 Jan 26;4(3):342–53. doi: 10.1002/cam4.374 (PMC4380960; doi:10.1002/cam4.374)
Supplement: Supplementary file 1 [file cam40004-0342-sd1.docx]

**Supplementary Information**

To validate the microarray methylation data, bisulfite sequencing was used for two of the microarray probes targeting the downstream region of the CDKN2A locus. Region 1 looked at cg10895543 (along with 5 other CpG sites) and primer set 1 was used. Region 2 looked at cg07752420 (along with 7 other CpG sites) and used primer set 2. MethPrimer (see below for citation) was used to generate PCR primers for bisulfite converted DNA (primer sequences listed below). 12 HPV- and 10 HPV+ tumor samples were analyzed (22 samples in total).

Primers Targeting the CpG from cg10895543 (Region 1):

Forward: 5’- TTTTTGAAATAAAATGGATGTTTAT -3’

Reverse: 5’- AAATACAAATACTCCCTCAAAAATA -3’

Tm=56.8°C

Product Size: 164bp

Primers Targeting the CpG from cg07752420 (Region 2):

Forward: 5’- TTTAGGTATTTTTTGTATTTGGTG -3’

Reverse: 5’- ACATCCATTTTATTTCAAAAATAAT -3’

Tm=58.1°C

Product Size: 167bp
